# Incidence of Facial Nerve Palsy in Pregnancy

**DOI:** 10.7759/cureus.31426

**Published:** 2022-11-12

**Authors:** Sushrut Bose, Ashish Anjankar

**Affiliations:** 1 Medicine, Jawaharlal Nehru Medical College, Datta Meghe Institute of Medical Sciences, Wardha, IND; 2 Biochemistry, Jawaharlal Nehru Medical College, Datta Meghe Institute of Medical Sciences, Wardha, IND

**Keywords:** bell's palsy, hemolysis, pregnancy, facial nerve paralysis, pre-eclampsia

## Abstract

The facial nerve is cranial nerve number seven. A facial nerve palsy is a form of severe weakness of muscles of the face or paralysis due to swelling or any other kind of injury to the seventh nerve. Bell’s palsy is the most common reason for facial nerve palsy in both pregnant and non-pregnant women, as well as in men and children. Palsy of the facial nerve is increased in conditions like pregnancy, diabetes, myasthenia gravis, Lyme disease, and multiple sclerosis, among others. The frequency of Bell’s palsy in pregnant women is about three times that of an average person. There are several theories that have not been proven as to why this is the case. They are elevation of clotting factors in the blood, total body water causing compression and swelling of the facial nerve, increased level of female hormones like estrogen and progesterone, and the weakening of immune system in the third trimester of pregnancy, leading to reactivation of many viruses. Also, Bell’s palsy during pregnancy has been linked to pre-eclampsia, which is also referred to as pregnancy-induced high blood pressure. Other associated factors are hemolysis, low platelets, which is a variant of pre-eclampsia, and elevated liver enzymes. Women are also susceptible to paralysis after childbirth. Most of the women who develop Bell’s palsy after pregnancy start showing signs and symptoms seven to 10 days after the date of delivery.

## Introduction and background

The facial nerve originates from the pons and passes through the ear to end up in the parotid gland. It is a mixed nerve, which means that it has both sensory and motor root. The former part is also known as the nerve of Wrisberg. The facial nerve is formed of a motor root (containing motor fibers to muscles of the face) and the nervus intermedius. The nervus intermedius contains sensory fibers for testing and parasympathetic fibers for lacrimal and salivary glands [1,2]. The nerve course is divided into four segments: intracranial, intrameatal, intratemporal, and extratemporal. The intratemporal segment is subdivided into 3A, 3B, and 3C, which are the labyrinthine segment, tympanic segment (also known as horizontal segment), and mastoid (also known as vertical segment). The part of the facial nerve in the fallopian (fascial) canal is the longest bony course among cranial nerves. The eighth nerve accompanies the facial nerve in the auditory meatus. The first and second genu are present in the tetra temporal segment of which the first genu has geniculate ganglion [3,4]. Branches of facial nerve supply various areas of the face and some around the face. There are no branches in segments 1, 2, and 3A. Three branches arise from the first genu, which is the greater superficial petrosal nerve (GSPN), which carries preganglionic parasympathetic fibers, lesser petrosal nerve, and external petrosal nerve [3,5]. The Vidian nerve, which is also known as the nerve of the pterygoid canal, is formed when the first preganglionic parasympathetic fibers of the GSPN pass through the petrotympanic fissure and join with the sympathetic fibers of deep petrosal nerve in the fossa lying in the middle part of the cranium. It then goes to the pterygopalatine (sphenopalatine) ganglion in the pterygopalatine fossa, of which postganglionic fibers come out and supply lacrimal glands, nasal glands, minor salivary glands and taste in the palate [4]. There are no branches for 3B. Just after the second genu, the facial nerve gives a branch which is the nerve to stapedius, which in turn is the first motor branch of the facial nerve. Before the facial nerve goes out of the stylomastoid foramen, it gives the first branch to develop embryonically, the chorda tympani nerve. It comes in from the posterior wall and comes out from the anterior wall through the canal of the Hugier and joins the lingual nerve, which gives nerve supply to the submandibular and sublingual salivary glands and taste in the anterior two-thirds of the tongue [3,4,6,7]. After coming out of the stylomastoid foramen, the facial nerve goes into the parotid gland. It divides the parotid gland into lobes, two in number, namely, the superficial lobe and the deep lobe. In the parotid gland, the facial nerve divides into five ending branches, namely, temporal, zygomatic, buccal, marginal mandibular, and cervical. Bill’s bar is an important surgical landmark of facial bone. It is a vertical ledge of bone. The facial nerve is 2.5 cm deep and internal to the cartilaginous tragal pointer. The facial nerve is anterior and superior to the digastric muscles. The facial nerve is superficial and anterior to the styloid process [2,5,8].

Branching pattern of facial nerve

Davis and Katz were the ones who described the anatomical branching of seventh nerve in their studies. The description given by Davis et al. stated that there were six branching patterns: type 1, in which the the branches of the facial nerve had no anastomoses between them; type 2, where the branches of temporofacial part have anastomosis; type 3, where the cervicofacial and temporofacial parts are connected to each other by anastomosis; type 4, which is both type 2 and type 4 combined; type 5, in which the cervicofacial part gives out two anastomotic rami that go and join with the temporofacial part; and lastly, type 6, which involves a plexiform pattern [9]. Katz and Catalano did a study by dissecting the parotid gland and gave five main types of patterns for the branching, which were: a straight branching pattern, known as type 1; loops involving zygomatic part and buccal part, known as types 2 and 3, respectively; a multiple interconnected complex arrangement, which is type 4; and lastly, type 5, in which a major and a minor trunk are present, both of which are the main trunks [10].

Electrophysiological testing

Usually, the stimulus is given proximal to injury and response is recorded distal to the stimulus. For facial nerve, a test is done after 72 hours. Sometimes, the stimulus can be given proximal to injury and response is recorded distal to stimulus even in facial palsy [4,8].

## Review

Facial nerve palsy

Bell’s palsy is the most common cause of facial nerve palsy and can be caused by swellings or idiopathy. In the case of the middle ear swollen, the resulting pressure may affect the nerve. If there is development of a rash along with blisters in the mouth as well, it may be due to a condition known as Ramsay Hunt syndrome caused by the herpes virus [11]. It is idiopathic. The weakness of facial muscles reaches a maximum within 24 hours of the onset of the palsy. The effected ear becomes oversensitive to sound, and there changed taste and senses [12]. There are other conditions like Lyme disease and stroke, which may show the same signs and symptoms as facial nerve palsy, so that additional tests may be required. It may also result in damage to other cranial nerves resulting in a condition known as poly neuropathy [13]. It can also occur if there is exposure to extreme hypothermia or emotional stress resulting in ischemia (this type of ischemia is known as primary ischemia). Secondary ischemia is caused when the nerve is compressed, and its circulation is affected; edema and increased capillarity permeability cause fluid exudation. If the fallopian canal is narrow due to genetics, even a little bit of nerve compression makes it probable to damage. It usually happens in people whose families have a history of it. Changes in lymphocytes have also been observed [14]. Table 1 contains information about grades of facial nerve palsy which tells us about the extent to which the nerve is damaged, according to which the treatment modality is decided. The extent of weakness or paresis is decided by certain topodiagnostic tests.

**Table 1:** Grades of facial palsy The table is the author's own creation.

Grade	Description	Characteristics
1	Normal	Normal functioning
2	Mild dysfunction	Slight weakness
3	Moderate dysfunction	Obvious weakness
4	Moderately severe disease	Disfiguring face
5	Severe dysfunction	Asymmetrical face
6	Total paralysis	No movement

Facial nerve palsy in pregnancy

In a study, there were eight people who presented with Bell's palsy during pregnancy out of which six were in their last trimester of pregnancy [15]. Bell’s palsy was reported to be an indicator of possible pre-eclampsia or haemolysis, elevated liver enzymes, low platelet count (HELLP) syndrome [15,16]. Pre-eclampsia occurs during pregnancy as a complication. In pre-eclampsia, a pregnant woman is presented with high blood pressure along with increased protein levels, which are elicited by increased excretion of proteins in urine. It is seen in the second trimester. Other symptoms include decreased platelet count in the blood, problems with sight like diminishing vision, respiratory symptoms like shortness of breath, abdominal cramps, particularly in the upper areas, abnormal liver function tests, which are generally increased, and headache, which is usually not normal [17-19]. Usually, if a pregnant woman has high blood pressure chronically or is overweight, the risk of getting facial paralysis is more, especially in the third trimester of pregnancy. The possible theories as to why facial nerve palsy is increased in pregnancy are the systemic changes occurring in the body, which increase its risk [18,20]. The definite pathogenesis of blood pressure as an affecting factor has not yet been elucidated. The thrombosis of vasa nervorum of facial nerve resulting in neuropathy, which is ischemic in nature and in the facial area. Further studies have also shown macrophages, and, thus, the immune system playing a major role in development of blood pressure. Disorders like gestational hypertension, gestational diabetes mellitus and pre-eclampsia during pregnancy are said to have played some part in the pathogenesis of failure of blood and nutrient circulation in the placenta. These diseases are called late pregnancy associated disorders. The onset of Bell’s palsy during pregnancy occurs at the same time as these diseases, and the etiologies and mechanisms are quite similar as well. Most of the previously published reports have been solely based on clinical observations due to shortage of patients. This is a limiting factor in determining the strength of the conclusion regarding the relation between pre-eclampsia and facial palsy. A case report also concluded that the weakness of the facial muscles progressed to complete palsy just ten days after the onset with less satisfactory outcomes [19,21]. The prognosis of Bell’s palsy in pregnancy is not that good. The recovery rates in cases of complete paralysis of facial muscles have been lesser than those of normal people. According to global statistics, the recovery rate is 52% in pregnant women, while it is 77-88% in women who are not pregnant and in the same age group [22,23]. Facial palsy, which is bilateral, is mostly rare but more commonly occurs during the final trimester of pregnancy. Bilateral facial nerve palsy is not commonly idiopathic, unlike the unilateral variant of it. There have been various hypotheses where different types of etiologies have been suggested for the bilateral type of palsy. Unilateral facial nerve palsies are primarily expected in the immediate post-partum period (time after the birth of the neonate). Bilateral palsy is the one that maximally occurs due to systemic changes in the body during pregnancy [24]. Bell’s palsy occurs most commonly in two age groups, mainly less than 40 years of age and above 60 years of age. The reason why it occurs at this younger age is suspected to be due to its increased occurrence during pregnancy. It is estimated to have an incidence of about eleven to forty in every one hundred thousand people [25].

Effects of facial nerve palsy in pregnant women

Bell’s palsy is the most common disorder causing mononeuropathy, which refers to a condition in which only a single nerve is affected. It is also the most common disorder associated with facial nerve weakness or paresis, or complete paralysis [26]. Other diseases of the facial nerve causing paresis include Millard Gilbert syndrome, a lesion around the sixth nerve nucleus along with the seventh nerve nucleus and facial nerve [3]. It is rapid in onset and affects the face unilaterally, which is presented as loss of facial expressions on one side of the face. It also causes inability or difficulty to close the eyelids due to loss of control of these muscles. This causes dryness of the eyes and, hence, leads to corneal pathologies resulting from the primary symptoms as blinking reflex is absent since facial nerve forms motor component of the reflex pathway. Mostly, recurrent infections occur in the patient. Long-term effects of this type of palsy are devastating to the patient. These signs also have importance in diagnosis of the disease in all the three trimesters of pregnancy, but mostly in the third trimester [26,27]. Since one side of the face is entirely non-functional, it leads to oral incompetence. This makes it difficult to masticate the food, making eating or chewing difficult. The person cannot even hold the contents in the mouth properly. The patients are usually advised to take in smaller morsels at a time. The paralysis also makes speaking very hard, and there is difficulty in pronouncing the words correctly and hence, understanding what they say is hard to comprehend [28,29].

Social impact of Bell's palsy

The psychological effects of this disorder are more than the physical disabilities. With the paralysis, people may refrain from taking photos as this makes them conscious. Bell’s palsy is also said to have made mothers not express their emotions towards their kids in a way they normally would. This is a time mixed with happiness and anxiety, plus the mother feels that she cannot help herself [28-30].

Treatment of Bell's palsy

Therapy with steroids usually involves oral corticosteroids, for example, prednisolone. It is most effective and starts within 72 hours of the onset of the palsy. This type of treatment is usually not approved by everyone since it may be detrimental to the health of the foetus. Prednisolone, when combined with other drugs like acyclovir etc., has not proven to be much beneficial as compared to prednisolone alone. If any kind of improvement does not occur and symptoms worsen further, then more investigations are done to find out in case it is a tumor of some sort [30-33]. If there is no recovery after four weeks of treatment, then the patients are advised to seek a specialist center's help. In most cases, patients with complete paralysis recover within six months, but in 30% of cases, this does not happen. We mostly treat the root causes like diabetes, obesity, etc., which are etiological factors [33-35]. A physical therapist gives exercises to cure the symptoms of paralysis. The primary importance is to prevent any ocular damage. The eye is exposed due to the absence of blinking reflex, which can cause dryness. So artificial tears are to be regularly applied to keep the eye lubricated. If one side of the face is paralyzed, then the exercises are given so that the person can move both sides of the face at least to some extent using innervation of only one side. The exercises will strengthen the muscles as we have to think of aspects of the muscle movement that we never had to worry about before. It might be more difficult for some patients to move their faces after recovery making them fear that the paralysis is returning but recurrence of this type of palsy is uncommon [36,37]. Table 2 contains information about the drugs that are conventionally used and their proven effectiveness in the treatment of Bell’s palsy.

**Table 2:** Management of Bell’s palsy The table is the author's own creation.

Medication	Category	Recommendation	Oral dose
Prednisolone	Corticosteroids	Proven effective	1 mg per kg body weight for 10 days
Acyclovir	Antivirals	Possibly effective	400 mg for 7 days

## Conclusions

Bell’s palsy, or facial nerve palsy, on both sides is very uncommon during pregnancy. If it occurs, it may be the primary sign of pre-eclampsia, which may be indicative of worse prognosis of the disorder. Hence, it requires more care and surveillance. This is not only for the treatment of the mother and the birth of the neonate but also to prevent the social or psychological effects during this course. Herpes virus, according to some doctors, is the most common cause, especially during pregnancy. The long-term outcomes of facial palsy in pregnant women were compared with women who were in their viable periods but were not pregnant at that time by blinded experts using the facial grading systems. These studies showed that the outcomes could not solely be explained by physical or medical therapies, mostly of the cases which occurred during the postpartum period as some major changes occur in the body, which cause complications like postpartum haemorrhage that are usually not present in other women of the same age group. It is important to note that cases which have been treated earlier than others showed lesser symptoms and signs of the disease, not only in Bell’s palsy cases but also in various other disorders involving the cranial nerves as the time the damaged nerve is affected plays a significant role in the reaction to injury, the primary reason for this being that the duration in which the nerve experiences the exposure to the causing factors increases and, therefore, damages the nerve more. Hence, to conclude, we cannot give any scientific evidence or proof as to why it is increased in pregnancy but we surely can put forward lots of hypotheses.

## References

[1] Kochhar A, Larian B, Azizzadeh B (2016). Facial nerve and parotid gland anatomy. Otolaryngol Clin North Am.

[2] Yang SH, Park H, Yoo DS, Joo W, Rhoton A (2021). Microsurgical anatomy of the facial nerve. Clin Anat.

[3] Monkhouse WS (1990). The anatomy of the facial nerve. Ear Nose Throat J.

[4] Proctor B (1991). The anatomy of the facial nerve. Otolaryngol Clin North Am.

[5] Lacombe H (2009). Functional anatomy of the facial nerve (Article in French). Neurochirurgie.

[6] Condie D, Tolkachjov SN (2019). Facial nerve injury and repair: a practical review for cutaneous surgery. Dermatol Surg.

[7] Guntinas-Lichius O, Eisele DW (2016). Facial nerve monitoring. Adv Otorhinolaryngol.

[8] Lorch M, Teach SJ (2010). Facial nerve palsy: etiology and approach to diagnosis and treatment. Pediatr Emerg Care.

[9] Davis RA, Anson BJ, Budinger JM, Kurth LR (1956). Surgical anatomy of the facial nerve and parotid gland based upon a study of 350 cervicofacial halves. Surg Gynecol Obstet.

[10] Katz AD, Catalano P (1987). The clinical significance of the various anastomotic branches of the facial nerve. Report of 100 patients. Arch Otolaryngol Head Neck Surg.

[11] Stuzin JM, Rohrich RJ (2020). Facial nerve danger zones. Plast Reconstr Surg.

[12] Owusu JA, Stewart CM, Boahene K (2018). Facial nerve paralysis. Med Clin North Am.

[13] Agostini F, Mangone M, Santilli V, Paoloni M, Bernetti A, Saggini R, Paolucci T (2020). Idiopathic facial palsy: umbrella review of systematic reviews and meta-analyses. J Biol Regul Homeost Agents.

[14] Foirest C, Bihan K, Tankéré F (2022). Peripheral facial palsy following COVID-19 vaccination: a practical approach to use the clinical situation as a guide. Acta Otorhinolaryngol Ital.

[15] Danielides V, Skevas A, van Cauwenberge P, Vinck B, Tsanades G, Plachouras N (1996). Facial nerve palsy during pregnancy. Acta Otorhinolaryngol Belg.

[16] Hussain A, Nduka C, Moth P, Malhotra R (2017). Bell's facial nerve palsy in pregnancy: a clinical review. J Obstet Gynaecol.

[17] McGregor JA, Guberman A, Amer J, Goodlin R (1987). Idiopathic facial nerve paralysis (Bell's palsy) in late pregnancy and the early puerperium. Obstet Gynecol.

[18] Evangelista V, Gooding MS, Pereira L (2019). Bell's palsy in pregnancy. Obstet Gynecol Surv.

[19] Leelawai S, Sathirapanya P, Suwanrath C (2020). Bell's palsy in pregnancy: a case series. Case Rep Neurol.

[20] Fuzi J, Spencer S, Seckold E, Damiano S, Meller C (2021). Bell's palsy during pregnancy and the post-partum period: a contemporary management approach. Am J Otolaryngol.

[21] Dorjey Y (2022). Bell's palsy with preeclampsia in pregnancy. Clin Case Rep.

[22] Choi HG, Hong SK, Park SK, Kim HJ, Chang J (2020). Pregnancy does not increase the risk of Bell's palsy: a national cohort study. Otol Neurotol.

[23] Phillips KM, Heiser A, Gaudin R, Hadlock TA, Jowett N (2017). Onset of bell's palsy in late pregnancy and early puerperium is associated with worse long-term outcomes. Laryngoscope.

[24] Ragupathy K, Emovon E (2013). Bell's palsy in pregnancy. Arch Gynecol Obstet.

[25] Leelawai S, Suwanrath C, Pruphetkaew N, Chongphattararot P, Sathirapanya P (2021). Gestational Bell's palsy is associated with higher blood pressure during late pregnancy and lower birth weight: a retrospective case-control study. Int J Environ Res Public Health.

[26] Baugh RF, Basura GJ, Ishii LE (2013). Clinical practice guideline: Bell's palsy. Otolaryngol Head Neck Surg.

[27] Fawale MB, Owolabi MO, Ogunbode O (2010). Bell's palsy in pregnancy and the puerperium: a report of five cases. Afr J Med Med Sci.

[28] Eviston TJ, Croxson GR, Kennedy PG, Hadlock T, Krishnan AV (2015). Bell's palsy: aetiology, clinical features and multidisciplinary care. J Neurol Neurosurg Psychiatry.

[29] de Sanctis Pecora C, Shitara D (2021). Botulinum toxin type A to improve facial symmetry in facial palsy: a practical guideline and clinical experience. Toxins (Basel).

[30] Heckmann JG, Urban PP, Pitz S, Guntinas-Lichius O, Gágyor I (2019). The diagnosis and treatment of idiopathic facial paresis (Bell's palsy). Dtsch Arztebl Int.

[31] Manthou ME, Gencheva D, Sinis N (2021). Facial nerve repair by muscle-vein conduit in rats: functional recovery and muscle reinnervation. Tissue Eng Part A.

[32] Shokri T, Saadi R, Schaefer EW, Lighthall JG (2020). Trends in the treatment of Bell's palsy. Facial Plast Surg.

[33] Tiemstra JD, Khatkhate N (2007). Bell's palsy: diagnosis and management. Am Fam Physician.

[34] Teixeira LJ, Valbuza JS, Prado GF (2011). Physical therapy for Bell's palsy (idiopathic facial paralysis). Cochrane Database Syst Rev.

[35] Badil Güloğlu S, Yalçın Ü (2021). Comparison of effects of low-level laser therapy and extracorporeal shock wave therapy in calcaneal spur treatment: a prospective, randomized, clinical study. Turk J Phys Med Rehabil.

[36] Burelo-Peregrino EG, Salas-Magaña M, Arias-Vázquez PI (2020). Efficacy of electrotherapy in Bell's palsy treatment: a systematic review. J Back Musculoskelet Rehabil.

[37] Pereira LM, Obara K, Dias JM, Menacho MO, Lavado EL, Cardoso JR (2011). Facial exercise therapy for facial palsy: systematic review and meta-analysis. Clin Rehabil.

